# Albumin-Based Nanoparticles for the Delivery of Doxorubicin in Breast Cancer

**DOI:** 10.3390/cancers13123011

**Published:** 2021-06-16

**Authors:** Rama Prajapati, Eduardo Garcia-Garrido, Álvaro Somoza

**Affiliations:** Instituto Madrileño de Estudios Avanzados en Nanociencia (IMDEA Nanociencia), Faraday 9, 28049 Madrid, Spain; rama.prajapati@imdea.org (R.P.); eduardo.garcia@imdea.org (E.G.-G.)

**Keywords:** bovine serum albumin (BSA), N-succinimidyl 3-(2-pyridyldithio) propionate (SPDP), doxorubicin (Dox), breast cancer, cross-linker

## Abstract

**Simple Summary:**

Doxorubicin (Dox) is a chemotherapeutic agent usually employed for the treatment of breast cancer. However, its use is limited because of the toxicities associated, and hence a proper delivery vehicle is necessary. In this sense, albumin-based nanocarriers are in the limelight for the successful delivery of chemotherapeutics because of safety, biocompatibility, and specific cancer-targeting properties. Herein, we have developed a nanocarrier system based on albumin, which selectively affects the tumor cells, and hence can revolutionize breast cancer therapy. The developed nanoparticles could be further used for the delivery of other hydrophobic drugs like SN38, which broadens the use of this system in the treatment of breast cancer.

**Abstract:**

Albumin-based nanoparticles are an emerging platform for the delivery of various chemotherapeutics because of their biocompatibility, safety, and ease of surface modification for specific targeting. The most widely used method for the preparation of albumin nanoparticles is by desolvation process using glutaraldehyde (GLU) as a cross-linker. However, limitations of GLU like toxicity and interaction with drugs force the need for alternative cross-linkers. In the present study, several cross-linking systems were evaluated for the preparation of Bovine Serum Albumin (BSA) nanoparticles (ABNs) encapsulating Doxorubicin (Dox). Based on the results obtained from morphological characterization, in vitro release, and therapeutic efficacy in cells, N-succinimidyl 3-(2-pyridyldithio) propionate (SPDP)-modified ABNs (ABN-SPDP) was chosen. Since ABN-SPDP are formed with disulfide linkage, the drug release is facilitated under a highly reducing environment present in the tumor sites. The cytotoxicity studies of those ABN-SPDP were performed in three different breast cell lines, highlighting the mechanism of cell death. The Dox-encapsulated ABN-SPDP showed toxicity in both the breast cancer cells (MCF-7 and MDA-MB-231), but, remarkably, a negligible effect was observed in non-tumoral MCF-10A cells. In addition to the hydrophilic Dox, this system could be used as a carrier for hydrophobic drugs like SN38. The system could be employed for the preparation of nanoparticles based on human serum albumin (HSA), which further enhances the feasibility of this system for clinical use. Hence, the albumin nanoparticles developed herein present an excellent potential for delivering various drugs in cancer therapy.

## 1. Introduction

Doxorubicin (Dox) is a widely used antineoplastic agent against various cancers, including breast cancer, Kaposi’s sarcoma, osteosarcoma, esophageal carcinoma, and Hodgkin’s and non-Hodgkin’s lymphomas [1]. It has been practiced in oncology since the late 1960s. However, the use of Dox is limited because of the increased toxicities, especially cardiotoxicity, mainly in those patients requiring dose escalations [1,2]. To enhance the therapeutic efficacy while decreasing the side effects, Dox was encapsulated in nanoparticles (NPs). In addition, the nanoparticles facilitate the passive tumor targeting through enhanced permeation and retention (EPR) effect and overcome the problems associated with multidrug resistance [3,4].

In this regard, NPs based on albumin offer various advantages, including biocompatibility, safety, and convenient surface modification due to the presence of carboxylic and amino groups [5,6]. Albumin is the most abundant protein in human blood, with a molecular weight of around 67 kDa and a circulation half-life of approximately 19 days [6]. In the body, it acts as a carrier for various compounds, including bilirubin, metal ions like zinc and copper, and it helps in the solubilization and transportation of hydrophobic long-chain fatty acids [7,8]. The development of albumin-based nanocarriers for drug delivery is interesting because of the various binding sites that facilitate incorporating hydrophilic and hydrophobic drugs in the particle matrix [9].

The tumoral uptake of albumin is enhanced by various factors, for instance, the higher concentration of albumin in blood than in the interstitial compartments allows the diffusion of albumin to tumor sites [10,11]. Moreover, various albumin receptors, namely, gp60 and SPARC, are overexpressed in cancer cells, which can contribute to enhance further the uptake of albumin-based cargos in the cancer sites [12]. For this reason, several approaches explore the use of albumin-based NPs for encapsulating Dox and increase the selective targeting to the tumor sites [13,14]. 

Regarding the preparation of this type of nanostructure, various techniques are available, like desolvation, emulsification, nano spray drying, and NAB-technology. Among them, the most widely used method is desolvation using ethanol. In those cases, to increase the stability of the final nanostructure, a cross-linking agent, such as glutaraldehyde, is usually employed. However, the limitations associated with glutaraldehyde, including toxicity, interaction with the encapsulated drug, and presence of residual aldehyde, limit the in vivo applications [15]. Therefore, stabilization strategies for albumin NPs are being searched for [16,17]. For instance, the use of glutathione to reduce the intramolecular disulfide bonds and stabilization of NPs by forming the intermolecular disulfide bonds was reported in previous studies [15,18]. Moreover, the surface of albumin can be modified with Traut’s reagent to introduce sulfhydryl groups, which can be further used for the conjugation of other moieties and has also been evaluated previously. 

Breast cancer is one of the most common types of cancer with an increasing incidence. Hormone receptor-positive and human epidermal growth factor receptor 2-negative breast cancer represent the largest subtype of this neoplastic disease [19]. For the treatment of these cancers, in the present study, bovine serum albumin (BSA) nanoparticles (ABNs) were prepared by desolvation method using ethanol. Herein, different stabilizing agents were evaluated, such as the cross-linking systems containing a disulfide bond to ease the release of the drugs inside the cells. Thus, the effect of various cross-linkers on drug loading, size, surface charge, and nanoparticular yield was studied. Finally, in vitro release studies and cell studies in MCF-7 and MDA-MB-231 breast cancer cells were performed.

## 2. Materials and Methods

### 2.1. Materials

All the solvents and chemical reagents were purchased from Sigma-Aldrich (San Luis, MO, USA), abcr GmbH (Karlsruhe, Germany), Thermo Fisher Scientific (Waltham, MA, USA), Scharlab (Sentmenat, Barcelona, Spain), FluoroChem (Hadfield, UK), and VWR (Radnor, PA, USA). Modified Eagle Medium (DMEM), streptomycin–penicillin (100X), fetal bovine serum (FBS), L-glutamine (100X), trypsin (10X), phosphate-buffered saline (PBS), and cell culture plasticware were purchased from VWR.

### 2.2. Preparation of BSA Nanoparticles (ABNs)

ABNs were prepared by the desolvation method, with ethanol as the desolvating agent. Depending upon the reagents used for the cross-linking of BSA, the ABNs were formed either by amide or disulfide bonds or by electrostatic stabilization.

#### 2.2.1. Formation of Amide Bonds

(a) Use of glutaraldehyde: For the preparation of nanoparticles using GLU (ABN-GLU), 20 mg/mL of BSA in an aqueous solution was incubated with 0.5 mg/mL doxorubicin Hcl for 2 h at room temperature. To this solution, 2.7 mL of anhydrous ethanol was added dropwise with the syringe pump at the constant flow rate of 1 mL/min. After the solution became turbid, 7 µL of 8% glutaraldehyde was added for cross-linking. The solution was stirred at 550 rpm for 18 h. Then, the free albumin, unbound Dox, ethanol, and excess glutaraldehyde, were removed by 3 cycles of centrifugations at 13,200 rpm for 15 min. After each centrifugation cycle, the pellets were redispersed in 1 mL of water.
(b) Use of EDC: For the preparation of ABNs using EDC (ABN-EDC), a freshly prepared aqueous solution of EDC (2 mg/mL) was added to the turbid solution of BSA after the desolvation process. The mixture was left rotating at 550 rpm for 3 h and purified by three cycles of centrifugation to remove the unreacted EDC and ethanol.

#### 2.2.2. Formation of Disulfide Bonds

(a) Use of glutathione: Firstly, the intramolecular disulfide bonds in albumin were cleaved by using glutathione (GSH), which is one of the major endogenous antioxidants in vivo. After the pre-treatment, it was purified using NAP-10 column and incubated with 0.5 mg/mL doxorubicin Hcl for 2 h, followed by desolvation with ethanol to precipitate albumin into Dox-loaded ABNs (ABN-GSH). Then, ethanol and unbound Dox were removed through centrifugation at 13,200 rpm for 15 min.
(b) Use of N-succinimidyl 3-(2-pyridyldithio) propionate (SPDP): The schematic representation for the preparation of ABNs by using SPDP (ABN-SPDP) is depicted in Figure S1. Firstly, thiol groups were introduced in BSA with 2-iminothiolane, commonly known as Traut’s reagent. In parallel, the same amount of BSA was modified with SPDP. The modified BSAs were purified with NAP-10 column and the resulting solutions combined. The mixture was incubated with DOX for 2 h, followed by preparation of NPs by desolvation method as described earlier in Section 2.2.1.
(c) Use of modified polyethylene glycol (PEG): The schematic representation of the synthesis of this derivative is provided in SI 2. The preparation of this modified PEG is as follows: to a solution of PEG(NH_2_)_2_ (3000 M.W.) (**1**) (100 mg, 33 µmol) in THF (6 mL) at 0 °C, a solution of SPDP (41.5 mg, 0.13 mmol) in THF (3 mL) was added. The reaction was allowed to warm up to room temperature under vigorous stirring for 16 h. Then, the solvent was removed under vacuum and re-dissolved in methanol (3 mL). The product was purified by dialysis using a 3.5 KDa. dialysis membrane for 16 h at 4 °C against distilled water. After this time, the solution turned cloudy, and the solvent was removed under vacuum. The desired product (**2**) was isolated as a greyish oil (44% of yield, 48.3 mg) (linker/polymer ratio 2:1). The product was characterized by NMR and MS (Figure S3). ^1^H NMR (D_2_O, 400 MHz): δ 8.26 (d, 2H), 7.71 (m, 4H), 7.17 (td, 2H), 3.56 (m, 264H), 3.21 (t, 4H), 2.93 (t, 4H). ^13^C NMR (D_2_O, 101 MHz): δ 173.74, 158.67, 149.03, 138.61, 121.69, 120.08, 38.83, 34.37, 33.56. MS (MALDI): theoretical mass: 3296.97, calculated mass: 3295.8.

Finally, the obtained product (**2**) was mixed with BSA modified with 2-iminothiolane at RT for 16 h, followed by incubation with Dox for 2 h and desolvation with ethanol to obtain the desired ABNs with modified PEG (ABN-PEG).

#### 2.2.3. Electrostatic Stabilization

For this approach, a polymer based on PEI and PEG, (PEI-PEG) was prepared through a three steps reaction. The synthetic process is schematically represented in Figure S2. The amine groups in 2 kDa linear PEI (**3**) were activated with 2-iminothiolane, commonly known as Traut’s reagent, to yield the derivative **4**, which contains the required thiols. Then, compound **4** was combined with the previously obtained compound **2**, and the mixture was stirred for 24 h at 400 rpm (at room temperature). The product was finally dialyzed against water to remove unreacted PEI, PEG, and 2-iminothiolane, leading to the desired compound **4**. (Figure S4). ^1^H NMR (400 MHz, D_2_O) δ 3.71 (s, 291 H), 3.27–2.21 (m, 305 H). ^13^C NMR (101 MHz, D_2_O) δ 174.07, 127.64, 121.90, 69.58, 68.81, 52.88, 51.03, 47.41, 45.52, 39.37, 39.02, 38.58, 37.55, 34.88, and 33.21. Finally, polymer **4** was incubated with 20 mg/mL BSA in water. The ABNs stabilized with PEI-PEG (ABN-PEI-PEG) and encapsulating DOX were then prepared, as discussed earlier in Section 2.2.1.

### 2.3. Surface Charge and Size Characterization of NPs

The Z-potential, size, and size distribution of all the prepared NPs were measured using a Zetasizer Nano ZS equipped with a 633 nm laser (Malvern Instruments, Worcestershire, UK). Prior to measurements at 25 °C and at 173° scattering angle, the samples were diluted 1/100 in water. ζ-potential was calculated by Zetasizer Software 7.11 (Malvern Instruments, Malvern, PA, USA) using Smoluchowski equation:

*μ_e_* = *ε_r_ε*_0_*ζ*/*η*

where *μ_e_* is the electrophoretic mobility, *ε_r_* is the dielectric constant of water, *ε*_0_ is the permittivity of vacuum, and *η* is the viscosity of the solvent (water). The measurements were done in triplicate and averaged.

### 2.4. Quantification of Nanoparticle Formation

To quantify the amount of BSA converted to nanoparticles, the Bradford protein assay was performed [20]. The calibration curve was made with the known concentrations of BSA standards. The nanoparticles were first separated by centrifugation at 13,200 rpm for 15 min at room temperature and the supernatant was analyzed spectrophotometrically at 595 nm. The nanoparticular yield was then calculated by using Equation (1) [21].

(1) $$NPs\;yield=\frac{Initial\;amount\;of\;BSA-amount\;of\;BSA\;in\;supernatant}{Initial\;amount\;of\;BSA}\times 100\%$$

### 2.5. Drug Loading of NPs

The amount of Dox incorporated in different ABNs was determined by an indirect method, i.e., by measuring the amount of the drug in the supernatant. The ABNs were separated by centrifugation, and the supernatant was analyzed spectrophotometrically at 495 nm. The drug loading capacity was then calculated following Equation (2).

(2) $$Drug\;loading=\frac{Dox\;added-amount\;of\;Dox\;in\;supernatant}{Dox\;added}\times 100\%$$

### 2.6. In Vitro Release Studies

The release of Dox from ABNs prepared by various methods was evaluated under the physiological pH of 7.4, an acidic pH of 5 adjusted with 0.1% acetic acid, and/or presence of 1 mM glutathione (GSH) to mimic the intracellular environment of tumor sites. At various time points, the aliquots of the sample were centrifuged at 13,200 rpm for 15 min at room temperature, and the supernatant was analyzed spectrophotometrically at 495 nm. The drug release was then calculated from a standard calibration curve of the free Dox solution. 

### 2.7. Stability Studies

The prepared ABN-SPDP suspension was stored either at 4 °C or at room temperature. Their stability was estimated by comparing the changes in their hydrodynamic size, zeta potential, and drug release every week for 2 months.

### 2.8. Cell Culture

MCF-7 and MDA-MB-231 cells were grown in DMEM containing 10% FBS, 1% streptomycin-penicillin, and 1% L-glutamine in a humidified incubator with 5% CO_2_ and 95% air at 37 °C. MCF-10A cells were grown in HUMEC basal serum-free medium containing HUMEC supplement, bovine pituitary extract (BPE), and 1% streptomycin-penicillin. All the experiments were conducted before the cells reached 25 passages.

#### 2.8.1. Cytotoxicity Assay

The cell viability of the prepared ABNs in MCF-7, MDA-MB-231, and MCF-10A cells was determined by alamarBlue assay. The cells in 100 µL of DMEM medium with 10% FBS, 1% streptomycin-penicillin, and 1% L-glutamine were seeded in 96-well plate (5 × 10^3^ cells per well) at 37 °C in a Binder CB210 incubator (5% CO_2_). After the cells reached 60% confluency, ABNs prepared with different cross-linkers were added to each well. After 48 h of incubation, the medium was removed, and 150 µL of DMEM with 1% resazurin was added. The supernatant was removed after 4 h of incubation at 37 °C, and the fluorescence was measured at excitation and emission wavelengths at 550 nm and 590 nm, respectively. The cell viability after 48 and 72 h was calculated as follows:

(3) $$Cell\;viability=\frac{Sample\;data-data\;of\;resazurin\;solution}{Data\;of\;untreated\;cells-data\;of\;resazurin\;solution}\times 100\%$$

#### 2.8.2. In Vitro Transfection

Transfection assay was carried out to observe the internalization and release of Dox in MCF-7 and MDA-MB 231 cells. For this purpose, firstly, the cells were seeded on P-6 polystyrene tissue-culture plates. After the cells reached 60% confluency, they were treated with ABN-SPDP. After 24 h of incubation, the cells were washed twice with PBS and the cells were examined for fluorescence using Beckman Coulter Cytomics 500 Flow Cytometer (Beckman Coulter, Indianapolis, IN, USA). The acquired data were analyzed with FloJo^TM^ v10.7 software (Ashland, Wilmington, DE, USA). All these experiments were performed in the Flow Cytometry Service at the CNB-CSIC.

#### 2.8.3. Determination of Cell Cycle Phase

Cells were harvested in 6-well plates. When the cells reached a 60% confluency, they were treated with ABN-SPDP encapsulating Dox at various concentrations. After 48 h, the samples were trypsinized, fixed with ethanol 70% (*v*/*v*), washed with PBS, and centrifuged at 100× *g* for 5 min in an Eppendorf centrifuge 5804 R (Eppendorf, Hamburg, Germany). For each sample, 10 μg RNAse A and 20 μg propidium iodide (PI) were added. Then, the cell cycle analysis was performed using a flow cytometer.

#### 2.8.4. Determination of Induction of Apoptosis/Necrosis

Cells were seeded in 6-well plates. When the cells reached a 60% confluency, they were treated with ABN-SPDP encapsulating Dox at various concentrations. After 24 h, the supernatant was collected, and the cells were trypsinized, washed with PBS, and centrifuged at 100× *g* for 5 min in an Eppendorf centrifuge 5804 R (Eppendorf, Hamburg, Germany). The cells were suspended in 100 µL of 1X binding buffer followed by the addition of 10 µL Annexin V 1X and incubated at 4 °C in darkness for 15 min. In each sample, 380 µL binding buffer 1X and 10 µL propidium iodide 1 mg/mL were added before analyzing them in a flow cytometer.

#### 2.8.5. Study of Mechanism of Internalization

To analyze the mechanism of internalization of ABN-SPDP in cells, MCF-7 cells were seeded in a 96-well plate at a density of 5 × 10^3^ cells/well. After the cells reached 60% confluency, they were washed with PBS followed by the addition of either 150 μM genistein or 5 μg/mL filipin (inhibitors for caveolae-mediated endocytosis), or chlorpromazine (10 μg/mL, an inhibitor for clathrin-mediated endocytosis). After 2 h of adding the endocytosis inhibitors, the cells were washed thrice with PBS and treated with Dox-loaded ABN-SPDP. The cells were washed again with PBS after 4 h of treatment, and new DMEM was added. Alamarblue assay, as described in Section 2.8.1, was conducted to compare the effect of endocytosis inhibitors in the internalization of ABNs.

#### 2.8.6. Western Blot Analysis

To address the effect of nanoparticles on cell cycle arrest phase, p53 and cyclin B and E protein levels were evaluated. 2 × 10^5^ MCF-7 or MDA-MB231 cells per p6 well were seeded and allowed to adhere to the plate. After 72 h of culture, cells were washed with PBS1x and incubated with the different treatments (as indicated in Results) in fresh medium. After 24 h of incubation, cells were washed with PBS1x, collected, and lysed with lysis buffer plus protein inhibitors (10 mM Tris-HCl pH 7.5, 5 mM EDTA, 150 mM NaCl, 10% glycerol, 0.5% Triton X-100, 50 mM sodium fluoride, 30 mM sodium pyrophosphate, 1 mM sodium orthovanadate, and 1 mM phenylmethylsulfonyl) and cell extracts were incubated for 30 min at 4 °C in a tube rotator. After clearing the total cell lysate by centrifugation for 15 min at 16100× *g* and 4 °C, supernatants were stored at −80 °C. Total protein amount was quantified by Bradford assay (Bio-Rad). Twenty milligrams of protein samples were separated on 12% SDS-polyacrylamide gels under reducing conditions and transferred to 0.45 μm nitrocellulose membrane (GE Healthcare Life science). Detection of specific proteins was performed by Western blot, employing the corresponding antibodies as indicated in the Results section. After incubating the membranes with 5% BSA in TBS-T (0. 0.1% Tween-Tris buffered saline) for 1 h at room temperature, the blots were incubated overnight at 4 °C with the corresponding first antibody solution in 3% BSA in TBS-T. Conditions for each antibody are the following: anti-p53 (1:500; sc-263), anti-cyclin B1 (1:500; sc-245), anti-cyclin E (1:500; sc-377100), and anti-GAPDH (1:500; sc-47724). After three washes with TBS-T, blots were incubated with peroxidase-labelled anti-mouse (1:5000; sc-516102) with 3% BSA in TBS-T blocking solution for 1 h at room temperature. The blots were washed three times with TBS-T and membrane-bound antibody was detected with enhanced chemiluminescence detection reagent (Bio-Rad). All antibodies were purchased from Santa Cruz Biotechnology. Densitometry analysis was performed using Fiji software (ImageJ).

### 2.9. Statistical Analysis

The results are presented as the mean ± standard deviation (SD). The statistical analysis was performed in R Project for Statistical Computing (R-3.2.5) software (R Development Core Team, Vienna, Austria) [22]. One-way analysis of variance (ANOVA) was used to compare the mean value of each condition versus the control. Significant differences between the means were accepted when the p-value was lower than 0.05 (*), 0.01 (**), and 0.001 (***). When the statistical difference was observed, Tukey’s test was performed to compare the mean values by pairs.

## 3. Results

### 3.1. Preparation and Characterization of ABNs

**Figure 1:**
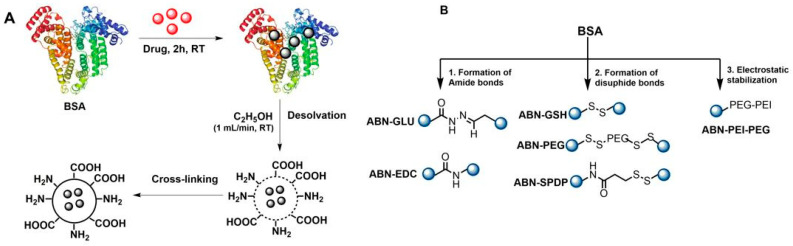
Schematic illustration of the preparation of ABNs. (**A**) Encapsulating a drug and (**B**) various approaches for the preparation of ABNs.

For the preparation of ABNs, BSA was incubated with the drug (e.g., Dox) for 2 h at RT, followed by the addition of ethanol at the rate of 1 mL/minute. The solution turns turbid due to the formation of ABNs, which are stabilized by the addition of cross-linkers. Depending on the cross-linker used, the mixture was incubated at RT for 3 h to 18 h. Then the samples were purified by centrifugation. (Figure 1A). The cross-linking agents employed (Figure 1B) lead to the different nanostructures of this study. For the ABNs stabilized with amide bonds, EDC or GLU was used. In the case of glutaraldehyde (GLU), the procedure was based on previous studies where ganciclovir was loaded onto ABNs [23]. GLU reacts with the amino groups on the surface of BSA and hence cross-links the BSA molecules with each other. On the other hand, with the use of EDC as a cross-linker, a peptide bond is formed between the carboxyl and amide groups of BSA particles [24]. It is important to note that the preparation time of ABNs was reduced to 3 h when EDC was used compared to overnight with GLU. Hence, it provides a rapid and straightforward technique for the preparation of ABNs.

Regarding the formation of disulfide bonds, three approaches were assessed. In the first one, BSA was treated with GSH to cleave any possible intramolecular disulfide moiety, followed by a solvation process, where the available thiols could be oxidized to yield the corresponding intermolecular disulfide bonds. In another approach, PEG containing activated disulfide groups was mixed with thiolated BSA to yield the desired ABNs. In the last approach involving disulfide bonds, an aqueous solution of BSA was modified with thiols, whereas another solution was treated with SPDP. Thus, the combination of both structures during the solvation process would yield a cross-linked structure due to the formation of disulfide bonds. Lastly, ABNs were also prepared where they were electrostatically stabilized with a tailor-made polymer based on PEI and PEG. In all the ABNs, the optimum concentration of Dox was chosen as 0.5 mg/mL.

**Figure 2:**
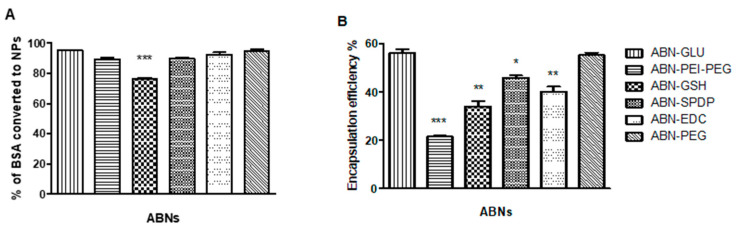
(**A**) Percentage of BSA converted to ABNs measured by Bradford assay and (**B**) Encapsulation efficiency of Dox in ABNs using various cross-linkers. Statistical analysis was performed using one-way ANOVA Tukey’s test. * *p*-value < 0.01, ** *p*-value < 0.001, and *** *p*-value < 0.0001.

The efficiency of the ABNs formulations was evaluated by the Bradford assay. In this case, the binding of the dye (Coomassie Brilliant Blue G-250) to protein causes the shift of maximum absorption of the dye from 465 to 595 nm, which could be used for the quantification of protein [20]. In all the cases, as shown in Figure 2A, more than 90% of the added BSA was converted to ABNs, except when GSH was used (75%). Next, the Dox encapsulated in each case was quantified using a UV-Vis spectrophotometer. In this regard, it is known that a variety of factors like the amount of drug added, type and amount of cross-linker used, and pH might affect the drug encapsulation [14]. In the present study, we focused on the effect of the different cross-linkers used in the preparation of ABNs on Dox loading. Particularly, the highest drug encapsulation (55%) was obtained using glutaraldehyde (GLU) as a cross-linker. On the other hand, when PEI-PEG was used to promote the formation of the nanostructure, the drug loading was the lowest (21%) (Figure 2B).

### 3.2. Size and Surface Charge Characterization of ABNs

**Figure 3:**
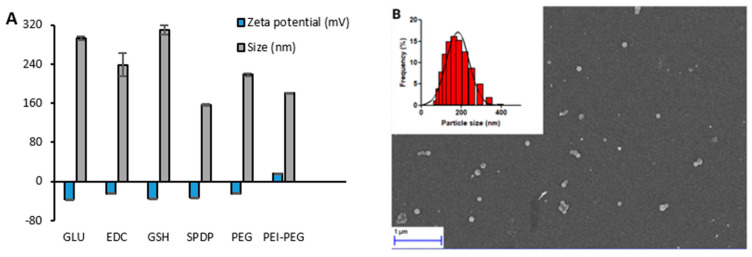
Size and zeta potential of Dox-loaded ABNs with various cross-linking methods (**A**); Size distribution of Dox-loaded ABN-SPDP observed with DLS and SEM (**B**).

It is important to gain insight into the size and surface charge characteristics of the prepared nanoparticles as they affect the colloidal stability, the nanoparticle cellular uptake, the pharmacokinetics, and the biodistribution after administration [25]. For instance, the absorption, biodistribution, metabolism, and excretion of nanoparticles are affected by their size and size distribution [26,27]. It was also found that the surface charge of nanoparticles could play an essential role in modulating the biodistribution and their cellular uptake and translocation [28]. As expected, the ABNs with PEI-PEG polymer had a positive surface charge (+15.4 ± 0.4mV). All other ABNs demonstrated a negative surface charge ranging from −24 ± 1.1 mV for ABN-EDC to −36.9 ± 0.3 mV for ABN-GLU. The size analysis demonstrated that ABN-SPDP had the smallest size of 156 ± 1.5 nm, whereas ABN-GSH had the largest size of 315 ± 4 nm (Figure 3A). The nanoparticles prepared with SPDP as cross-linker were further analyzed by SEM for morphology. The nanoparticles had a nanometric size and spherical morphology (Figure 3B). Based on the results from the drug loading, percentage of albumin converted to the NPs, and size characterization, the ABNs prepared with PEI-PEG and GSH were ruled out for further evaluation. Additionally, the ABNs prepared with PEG were also discarded since they were not stable for more than a week despite showing promising encapsulation efficiency of approximately 55%.

### 3.3. In Vitro Release of Dox from ABNs

For enhanced drug efficacy and specificity, the nanoparticles that can respond to particular conditions in the tumor cells, like changes in pH, enzyme activity profile, or redox state, are of great interest [29]. In this regard, pH-triggered drug release is one of the important parameters in cancer therapy since the extracellular microenvironment of tumor cells has acidic pH of around 5.5–6.5 compared to healthy cells with a pH of 7.4 [30,31]. This difference in pH of the target site may facilitate the specific drug targeting to the tumor sites. Moreover, solid tumors have elevated levels of glutathione (GSH), which can also be exploited to trigger the release of anticancer drugs [32,33,34,35]. In this regard, the disulfide bonds within the nanoparticles can be cleaved in the presence of GSH, enhancing the release of the drug [36].

**Figure 4:**
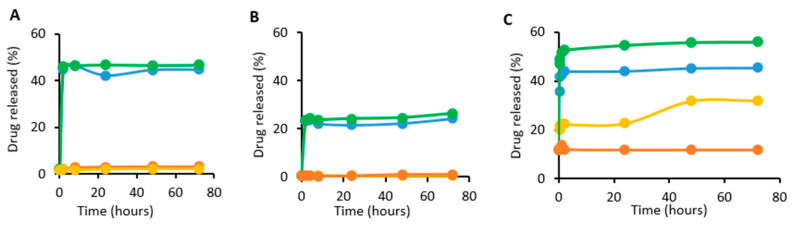
Release of Dox from ABNs with EDC (**A**), Glutaraldehyde (**B**), and SPDP (**C**) at different time points. The conditions tested were pH 5 (blue), GSH 1 mM (yellow), pH 5 and GSH 1 mM (green), and pH 7 with GSH 1 µM (orange).

Hence, the effect of using different cross-linkers on the release of encapsulated Dox was thus studied at acidic pH of 5 and/or in the presence of glutathione at different time points. As shown in Figure 4, approximately 56% of encapsulated Dox was released from ABN-SPDP after 72 h, whereas only around 26% was released from ABN-GLU. The release of Dox from ABN-EDC at the acidic pH was around 46% after 72 h. Moreover, the release of the drug was enhanced by the use of 1 mM GSH in the case of ABN-SPDP. However, the release was not affected in ABNs with glutaraldehyde and EDC.

### 3.4. Cell Viability Studies

**Figure 5:**
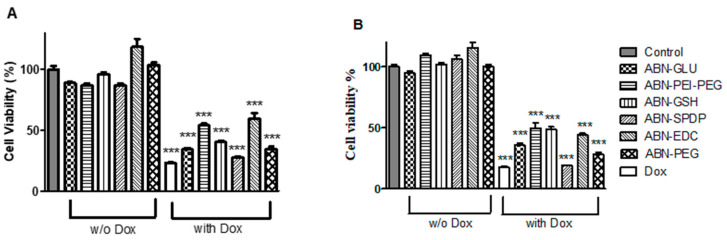
Cell viability assay of Dox-loaded ABNs with different cross-linkers in MCF-7 cells after 48 h (**A**) and 72 h (**B**) of treatment. In all the cases, the concentration of Dox used was 1 µM; 2–6: ABNs without Dox and 8–12: ABNs with Dox. Statistical analysis was performed using one-way ANOVA Tukey’s test. *** *p*-value < 0.0001.

The cell viability of MCF-7 cells 24- and 48-h post-treatment with ABNs was carried out by using alamarBlue assay. All the ABNs without Dox showed more than 95% cell viability, which indicates the possible use of these systems to deliver Dox to the tumor sites. When Dox-loaded ABNs were used, the highest number of cells were killed when ABN-SPDP were used, followed by ABN-PEG, ABN-GLU, ABN-EDC, ABN-PEI-PEG, and ABN-GSH, respectively (Figure 5).

Based on these results, SPDP was selected as an optimum cross-linker to produce ABNs encapsulating doxorubicin. The findings support the idea that SPDP can be used as a cross-linking agent, which can serve as a possible alternative method to toxic glutaraldehyde. Furthermore, to assess the scope of the approach, a different type of drug was also evaluated. In this case, the hydrophobic drug SN38 was encapsulated in ABN-SPDP, and the efficacy was analyzed in breast cancer cell lines. The drug concentration of up to 2 mg/mL could be loaded in these ABNs, which resulted in the particles with the size of 215.1 ± 7.3 nm and zeta potential of −25.5 ± 1.6 mV. The ABNs resulted in the reduction of cell viability to approximately 25% and 23% in MCF-7 and MDA-MB-231 cells, respectively, 48 h post-treatment (Figure S5). Thus, these results confirm the potential of the approach for the encapsulation, delivery, and controlled release of hydrophobic and hydrophilic drugs such as SN38 and DOX, respectively.

### 3.5. Stability Studies

**Figure 6:**
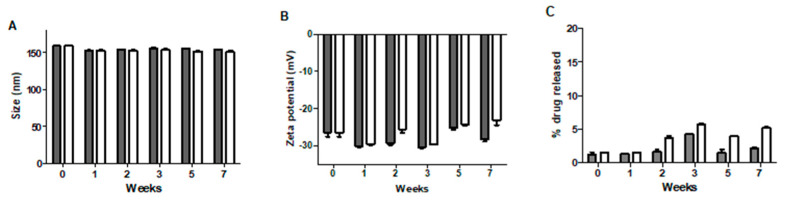
Change in size (**A**), zeta potential (**B**), and drug release (**C**) of Dox-loaded ABN-SPDP at different time points at two different temperature conditions, 4 °C (gray) and RT (white). Till the end of 2 months, the ABN-SPDP were stable. The data are presented as mean ± standard deviation (SD).

The long-term colloidal stability of nanoparticular suspension was evaluated since it could be critical for future applications. ABN-SPDP had pH of 8.04 and were stored either at room temperature or at 4 °C. The size distribution and surface potential of these nanoparticles were measured every week for 2 months. As seen in Figure 6A–C, the nanoparticles were stable and maintained colloidal stability at both temperatures during the period of the study. The DLS results were constant for the samples irrespective of the storage temperatures, and hence it can be concluded that the nanoparticles are stable at both the storage conditions. The great stability observed can be due to the repulsive forces between the particles, which might reduce the particle collisions, and hence prevent particle aggregation [37].

### 3.6. Cell Studies

**Figure 7:**
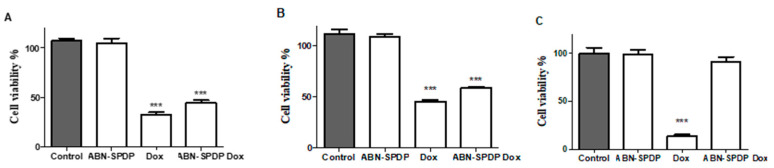
Cell viability assay with Dox-loaded ABN-SPDP 48 h post-treatment in (**A**)MCF-7 cells, (**B**) MDA-MB-231, and (**C**) MCF-10 A. In all the cases, the concentration of Dox used was 2 µM. Statistical analysis was performed using one-way ANOVA Tukey’s test (*** *p*-value < 0.0001).

The transfection efficiency of Dox-loaded ABN-SPDP was investigated in MCF-7 and MDA-MB-231 human breast cancer cells. The fluorescence of free Dox and encapsulated in ABN-SPDP was similar in both the cell lines (>98%) (Figure S6). Those ABNs were further tested in various cell lines (MCF-7, MDA-MB-231, and MCF-10 A). As shown in Figure 7A–C, the ABN-SPDP were not toxic in any of the cell lines. The cell viability was significantly decreased when free Dox or Dox-loaded ABN-SPDP were used in both MCF-7 and MDA-MB-231 cells. It is to be noted that when free Dox was administered in non-tumoral MCF-10 A cells, it was highly toxic, resulting in cell viability of around 20%. Remarkably, the cell viability when Dox-loaded in ABN-SPDP was administered was not significantly different from the untreated ones (Figure 7C).

### 3.7. Effect on Apoptosis/Necrosis of the Cells

A study was conducted to verify the effect of Dox-loaded ABN-SPDP in the apoptosis/necrosis of MCF-7 and MDA-MB-231 cells. As shown in Table 1, the percentage of healthy cells decreased in a dose-dependent manner in both the cell lines when treated with 1 µM, 2 µM, and 4 µM Dox-loaded ABN-SPDP. There was an increase in apoptotic cells by 1.43, 1.8, and 2.13 folds when MCF-7 cells were treated with 1 µM, 2 µM, and 4 µM Dox-loaded ABN-SPDP, respectively. Moreover, the percentage of necrotic cells also increased from 17.45% to 24.05% when the concentration of Dox-loaded ABN-SPDP was increased from 1 µM to 4 µM. A similar result was obtained with MDA-MB-231 cells. The percentage of apoptotic cells increased by 5.7, 6.9, and 7.5% when treated with 1 µM, 2 µM, and 4 µM Dox-loaded ABN-SPDP, respectively. Moreover, the necrotic cells increased to 33.9% when treated with 4 µM Dox-loaded ABN-SPDP. Hence, Dox is found to induce apoptosis and necrosis in both MCF-7 and MDA-MB-231 cell lines irrespective of the receptor present. 

### 3.8. Determination of Dominant Cell Cycle Phase

**Figure 8:**
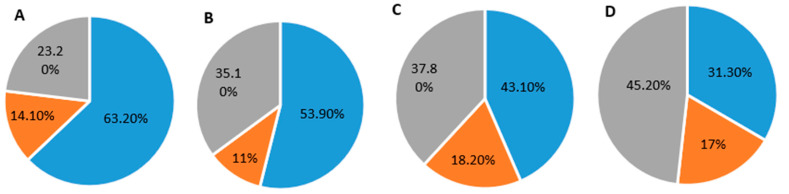
Percentage of cells in different phases of the cell cycle in MCF-7 cells and MDA-MB-231 cells. (**A**,**B**) Cells treated with 1 µM Dox and Cells treated with 1 µM Dox-loaded in ABN-SPDP in MCF-7 cells. (**C**,**D**) Cells treated with 1 µM Dox and Cells treated with 1 µM Dox-loaded in ABN-SPDP in MDA-MB-231 cells. Blue: cells in G0/G1 phase; orange: cells in S phase; grey: cells in G2/M phase.

The cell cycle assay was carried out to detect the dominant cell cycle phase in both MCF-7 and MDA-MB-231 cells. As shown in Figure 8, when MCF-7 cells were treated with 1 µM Dox alone or with Dox-loaded ABN-SPDP, the cells in cell cycle arrest (G0/G1) increased by 1.37 and 1.17 folds, respectively, compared to the empty ABNs, whereas G2/M phase cells elevated by 1.11 and 1.52 folds. On the other hand, when MDA-MB-231 cells were treated with 1 µM Dox or Dox-loaded ABN-SPDP, the number of cells in G2/M phase increased by 1.59 and 1.63 folds, respectively, compared to the empty ABN-SPDP. The cycle arrest was evident in both G0/G1 and G2/M phases in MCF-7 cells, while it was most prominent in G2/M phase in MDA-MB-231 cells. Oncul and co-workers obtained similar results when Dox was used in MCF-7 and MDA-MB-231 cells to study multidrug resistance and apoptosis [38].

### 3.9. Western Blot Analysis

Western blot assay was performed to assess the effect of free Dox and Dox-loaded ABN-SPDP on the expression of various proteins, namely, P53, cyclin B, and cyclin E. In the experiments, GAPDH with the molecular weight of 36 kDa was used as a control to assess the amount of protein loaded in each lane. As demonstrated in Figure S7, the levels of P53 were enhanced in MCF-7 cells when treated with free Dox or ABN-SPDP loaded with Dox. However, no change was observed in MDA-MB-231 cells. This difference in P53 activity in two different breast cancer cell lines is attributed to the difference in cell cycle arrest phases by Dox, which was demonstrated by the levels of different cyclins involved in cell cycles. The level of cyclin E, responsible for the cell cycle arrest in G0/G1 phase, was significantly enhanced in MCF-7 cells, while it was unchanged in MDA-MB-231 cells. However, the level of cyclin B, indicative of the cell cycle arrest in G2/M phase, was enhanced in both cell lines, most prominently in MDA-MB-231 cells, when treated with Dox-loaded ABN-SPDP. Hence, these results are in accordance with the observations from flow cytometry studies.

### 3.10. Determination of the Mechanism of Internalization of NPs

**Figure 9:**
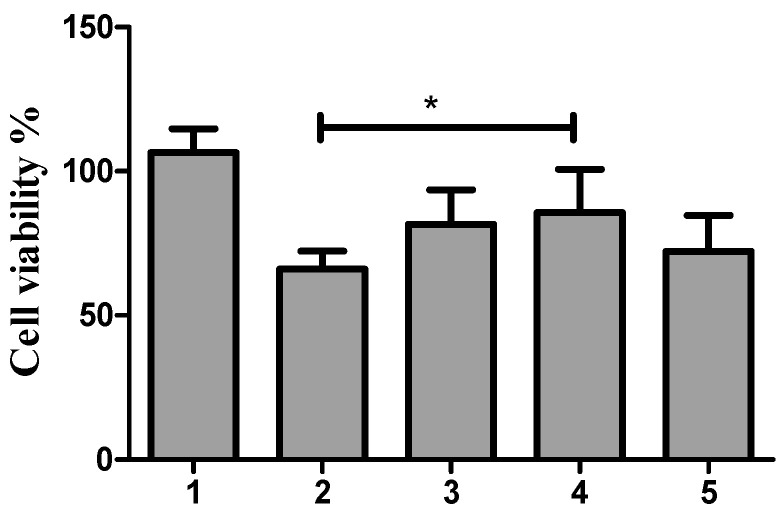
Cell viability assay with Dox-loaded ABN-SPDP in the presence of endocytosis inhibitors 24 h post-treatment in MCF-7 cells. 1: control, 2: ABN-SPDP Dox without endocytosis inhibitors, 3: in the presence of genistein, 4: filipin, and 5: chlorpromazine. Statistical analysis was performed using one-way ANOVA Tukey’s test (* *p*-value < 0.05).

There are various pathways through which cells can uptake the nanoparticles, including clathrin-mediated endocytosis; caveolae-mediated endocytosis; and clathrin- and caveolae-independent pathways. In the present study, the intracellular trafficking of Dox-loaded ABN-SPDP was investigated by using Genistein or Filipin (caveolae-mediated endocytosis inhibitors) or chlorpromazine (clathrin-mediated endocytosis inhibitor), and cell viability was measured after 24 h of treatment. As shown in Figure 9, the cell viability in MCF-7 cells was increased from 66% with Dox-loaded ABN-SPDP in the absence of any endocytosis inhibitors to 81% with genistein, 85% with filipin, and 72% with chlorpromazine. The obtained results indicate that the ABNs are internalized by both endocytotic pathways, with caveolae playing a more prominent role. These results are in agreement with a previous report where human serum albumin nanoparticles were used for gene delivery [39].

## 4. Discussion

Dox is a widely used anticancer drug that targets topoisomerase II and inhibits the replication and transcription of DNA, leading to cell cycle arrest and apoptosis [40]. Despite its therapeutic efficacy in various types of cancer including Kaposi’s sarcoma, osteosarcoma, and breast cancer, increased toxicity, particularly cardiotoxicity, limit its use [1]. Hence, the encapsulation of Dox in NPs was used to enhance therapeutic efficacy while reducing the off-target effects. In the present study, we developed a nanocarrier system based on albumin because of various advantages, including biocompatibility, safety, and the possibility of surface modification. For the preparation of ABNs, various techniques like desolvation, thermal gelation, emulsification, nano spray drying, and self-assembly can be used. However, desolvation is the most commonly used method, where ethanol is employed as a desolvating agent and glutaraldehyde as a cross-linker [39,41]. Unfortunately, using glutaraldehyde as a cross-linker has some disadvantages like toxicity, potential reaction with encapsulated drug, and longer reaction time to prepare albumin nanoparticles [17,42]. Hence, alternative cross-linkers and procedures are desirable to improve the properties of albumin-based nanostructures. 

Herein, we have developed and evaluated different linkers for the preparation of this type of nanostructure to encapsulate the chemotherapeutic drug Dox (Figure 1B). In the present study, three approaches were explored, which can be classified based on the bond generated to stabilize the nanostructure. Particularly, the formation of amide and disulfide bonds are explored here. Complementarily, the formation of nanostructures by electrostatic interactions is also studied. In the case of nanostructures stabilized by an amide bond (ABN-GLU and ABN-EDC), robust structures are expected, but the release of the chemotherapeutics might be limited. On the other hand, when disulfide bonds are used (ABN-GSH, ABN-PEG and ABN-SPDP), the release of the drugs can be promoted by glutathione present in the cells. Finally, the nanoparticles stabilized electrostatically (ABN-PEI-PEG) can be disassembled inside the cell due to the pH of lysosomes or ionic strength.

The size of NPs plays a vital role in determining the fate inside biological systems. It was observed that the NPs smaller than 6 nm are rapidly excreted by the kidney while those larger than 200 nm are accumulated in the spleen and liver [43]. In this sense, the ABN-SPDP with size of around 156 nm measured by DLS (Figure 3A), are optimal compared to other ABNs with size > 200 nm. In addition, the release of Dox from ABN-SPDP was highest in tumor-mimicked environments, i.e., with acidic pH and in the presence of glutathione (Figure 4C). This is because the ABNs are cross-linked with the disulfide bridges, and hence the higher concentration of GSH in the tumor cells causes destabilization of ABNs, leading to a higher release of drug. However, the use of GLU results in ABNs with a compact cross-linked matrix with smaller pores for drug diffusion that leads to lower drug release [14] (Figure 4A). This result was also observed in vitro in MCF-7 cells, where ABN-SPDP showed enhanced efficacy compared to the rest of ABNs, which was comparable to the free drug (Figure 6). Based on these results, SPDP was chosen as an optimum cross-linker.

The cell studies of ABN-SPDP were then conducted in breast cancer cells (MCF-7 and MDA-MB-231) and non-tumoral epithelial mammary cells (MCF-10A). The free Dox showed prominent toxicity in all the tumoral and non-tumoral cell lines, whereas when the drug was used encapsulated in ABN-SPDP, it showed significant toxicity only in the cancer cells (Figure 7A–C). This can be attributed to the fact that the release of drug from the ABNs employed is triggered by the acidic conditions and presence of glutathione, which is favorable only in the tumor cells and not in the non-tumoral mammary epithelial cells (MCF-10A). Hence, these formulations could be used to reduce the toxicity problems associated with the delivery of Dox and could revolutionize the treatment option in breast cancer therapy.

Furthermore, it was of interest to study the mechanism by which Dox affects the cells. We observed that both free Dox and encapsulated in ABN-SPDP showed a similar trend in terms of cell apoptosis and necrosis in both the cell lines (Table 1). However, there was a differential effect in cell cycle arrest in MCF-7 and MDA-MB-231 cells when treated with Dox (Figure 8A–D). This difference can be attributed to the variation in the p53 activity in two cell lines. MCF-7, an estrogen-dependent cell line, consists of the wild type p53, whereas MDA-MB-231, an estrogen-independent cell line, has p53 mutated and inactive [44,45]. The active P53 in MCF-7 cells after DNA damage mediated by Dox treatment can induce p21 upregulation responsible for cell cycle arrest in G0/G1 phase. In contrast, p53 levels in MDA-MB-231 cells are not affected by Dox treatment, and hence p21 levels do not change significantly and therefore induce the cell cycle arrest in G2/M phase [44]. This result was further supported by Western blot assays (Figure S7).

Finally, the feasibility of this approach for the preparation of nanostructures based on human serum albumin (HSA) was studied, which might be required for the clinical translation. HSA is particularly useful to avoid any immunological response in studies involving humans [46]. Remarkably, the HSA NPs prepared behaved similarly to ABN-SPDP with a similar size and zeta potential of 161.3 ± 4.6 nm and −31.2 ± 3.2 mV, respectively. Interestingly, the encapsulation efficiency of Dox was increased to 62.9% when HSA was used instead of BSA. The in-vitro studies conducted in MCF-7 and MDA-MB-231 cells revealed improved activity in both the cell lines. The cell viability was reduced to 37% and 51%, respectively, in MCF-7 and MDA-MB-231 cells when treated with Dox-loaded HSA NPs (Figure S8). Moreover, the cell cycle analysis in both the cell lines revealed elevated cell cycle arrest in G2/M phase (Figure S9). All these results obtained with HSA highlight the potential clinical translation of these nanocarrier systems.

## 5. Conclusions

In the present study, different cross-linking processes were employed to prepare ABNs loaded with Dox to improve encapsulation efficiency, the in-vitro release of the drug, and enhancement of activity in the selected cell lines. When SPDP was used as a cross-linker, the release of drug from ABNs was enhanced, demonstrated by in-vitro release in an acidic environment and in the presence of glutathione, and the activity in two different cell lines, MCF-7 and MDA-MB-231. Interestingly, the NPs could target only the tumor cells and spared non-tumoral cells, demonstrated by the experiments in MCF-10A epithelial mammary cells.

The results presented here contribute to the fundamental understanding of the preparation of the optimum ABNs for encapsulation of anticancer drug, Dox. Moreover, this approach could be successfully used for the preparation of HSA NPs, which can ease the translation of this system to clinics. These experimental findings could guide the development of advanced cancer nanostructures for targeted delivery.

## Figures and Tables

**Table 1:** Percentage of healthy, necrotic, and apoptotic cells with varying concentrations of Dox-loaded ABN-SPDP 24 h post-treatment in MCF-7 and MDA-MB-231 cells.

	MCF-7			MDA-MB-231		
	Healthy Cells	Apoptotic Cells	Necrotic Cells	Healthy Cells	Apoptotic Cells	Necrotic Cells
Control	99.45 ± 0.45	0.15 ± 0.05	0.10 ± 0.10	99.75 ± 0.15	0.2 ± 0.10	0.05 ± 0.10
ABNs	95.15 ± 0.85	1.40 ± 0.10	4.00 ± 0.20	95.75 ± 3.15	1.15 ± 0.15	3.65 ± 0.25
Dox alone 2 µM	55.45 ± 0.85	3.70 ± 0.20	18.40 ± 0.10	72.00 ± 0.50	6.20 ± 0.10	12.95 ± 0.35
ABN-SPDP Dox 1 µM	65.70 ± 0.40	2.15 ± 0.15	17.45 ± 1.05	69.20 ± 0.51	5.65 ± 0.05	14.80 ± 0.10
ABN-SPDP Dox 2 µM	60.90 ± 0.40	2.70 ± 0.10	20.90 ± 3.80	63.80 ± 0.48	7.15 ± 0.25	21.00 ± 0.30
ABN-SPDP Dox 4 µM	50.95 ± 6.35	3.20 ± 1.00	24.05 ± 4.65	44.50 ± 2.48	7.30 ± 0.20	33.90 ± 0.40

## Data Availability

The data presented in this study are available on request from the corresponding author.

## Supplementary Materials

The following are available online at https://www.mdpi.com/article/10.3390/cancers13123011/s1. Figure S1. Surface modification of BSA with Traut’s reagent and SPDP, followed by their conjugation through the formation of disulfide bonds to yield the corresponding BSA nanoparticles (ABN-SPDP). Figure S2. Schematic illustration of the preparation of PEI-PEG based polymer. Figure S3. ^1^H (A) ^13^C NMR (B) and MS (C) of NH_2_-PEG-NH_2_ modified with SPDP. Figure S4. ^1^H (A) and ^13^C NMR (B) of PEI-PEG-PEI. In ^1^H NMR, the PEG/PEI signal integrates in the ratio of 1:1.05, which corresponds approximately to the bi-functionalized PEG with PEI. In ^13^C NMR, the characteristics peaks of the PEG at 70 ppm, and the peaks of PEI distributed between 50 and 35 ppm can be observed that correspond to the ethylene groups. Figure S5. Cell viability assay with SN38 loaded ABN-SPDP in (A) MCF-7 and (B) MDA-MB-231 cells 48 h post-treatment. Statistical analysis was performed using one-way ANOVA Tukey’s test (*** *p*-value < 0.0001). Figure S6. Quantification of fluorescence of Dox using flow cytometry in MDA-MB-231 (A) and MCF-7 (B) cells 24 hours post-treatment. Figure S7. Western blot assay to assess the effect of Dox loaded ABN-SPDP on the expression of P53 and cyclins in MCF-7 and MDA-MB-231 cells. Figure S8. Cell viability assay with Dox loaded HSA NPs in (A) MCF-7 and (B) MDA-MB-231 cells 48 h post-treatment. Statistical analysis was performed using one-way ANOVA Tukey’s test (*** *p*-value < 0.0001). Figure S9. Percentage of cells in different phases of the cell cycle in (A) MCF-7 and (B) MDA-MB-231 cells treated with Dox-loaded HSA NPs. Blue: cells in G0/G1 phase; orange: cells in S phase; grey: cells in G2/M phase.

## References

[1] Singal P.K., Iliskovic N. (1998). Doxorubicin-induced cardiomyopathy. N. Engl. J. Med..

[2] Rivankar S. (2014). An overview of doxorubicin formulations in cancer therapy. J. Cancer Res. Ther..

[3] Shi Y., van der Meel R., Chen X., Lammers T. (2020). The EPR effect and beyond: Strategies to improve tumor targeting and cancer nanomedicine treatment efficacy. Theranostics.

[4] Kalyane D., Raval N., Maheshwari R., Tambe V., Kalia K., Tekade R.K. (2019). Employment of enhanced permeability and retention effect (EPR): Nanoparticle-based precision tools for targeting of therapeutic and diagnostic agent in cancer. Mater. Sci. Eng. C.

[5] Hoogenboezem E.N., Duvall C.L. (2018). Harnessing albumin as a carrier for cancer therapies. Adv. Drug Deliv. Rev..

[6] Larsen M.T., Kuhlmann M., Hvam M.L., Howard K.A. (2016). Albumin-based drug delivery: Harnessing nature to cure disease. Mol. Cell. Ther..

[7] Curry S., Mandelkow H., Brick P., Franks N. (1998). Crystal structure of human serum albumin complexed with fatty acid reveals an asymmetric distribution of binding sites. Nat. Struct. Biol..

[8] Kragh-Hansen U. (1981). Molecular Aspects of Ligand binding to serum albumin. Pharmacol. Rev..

[9] Patil G.V. (2003). Biopolymer albumin for diagnosis and in drug delivery. Drug Dev. Res..

[10] Evans T.W. (2002). Review article: Albumin as a drug—biological effects of albumin unrelated to oncotic pressure. Aliment Pharmacol. Ther..

[11] Kratz F. (2010). Albumin, a versatile carrier in oncology. Int. J. Clin. Pharmacol. Ther..

[12] An F., Zhang X. (2017). Strategies for Preparing Albumin-based Nanoparticles for Multifunctional Bioimaging and Drug Delivery. Theranostics.

[13] Onafuye H., Pieper S., Mulac D., Cinatl J., Wass M.N., Langer K., Michaelis M. (2019). Doxorubicin-loaded human serum albumin nanoparticles overcome transporter-mediated drug resistance. Beilstein J. Nanotechnol..

[14] Dreis S., Rothweiler F., Michaelis M., Cinatl J., Kreuter J., Langer K. (2007). Preparation, characterisation and maintenance of drug efficacy of doxorubicin-loaded human serum albumin (HSA) nanoparticles. Int. J. Pharm..

[15] Wang W., Huang Y., Zhao S., Shao T., Cheng Y. (2013). Human serum albumin (HSA) nanoparticles stabilized with intermolecular disulfide bonds. Chem. Commun..

[16] Amighi F., Emam-Djomeh Z., Labbafi-Mazraeh-Shahi M. (2020). Effect of different cross-linking agents on the preparation of bovine serum albumin nanoparticles. J. Iran. Chem. Soc..

[17] Niknejad H., Mahmoudzadeh R. (2015). Comparison of different crosslinking methods for preparation of docetaxel-loaded albumin nanoparticles. Iran. J. Pharm. Res..

[18] Zhao S., Wang W., Huang Y., Fu Y., Cheng Y. (2014). Paclitaxel loaded human serum albumin nanoparticles stabilized with intermolecular disulfide bonds. Medchemcomm.

[19] Silvestri M., Cristaudo A., Morrone A., Messina C., Bennardo L., Nisticò S.P., Mariano M., Cameli N. (2021). Emerging Skin Toxicities in Patients with Breast Cancer Treated with New Cyclin-Dependent Kinase 4/6 Inhibitors: A Systematic Review. Drug Saf..

[20] Bradford M.M. (1976). A rapid and sensitive method for the quantitation of microgram quantities of protein utilizing the principle of protein-dye binding. Anal. Biochem..

[21] Zhang S., Wang G., Lin X., Chatzinikolaidou M., Jennissen H.P., Laub M. (2008). Polyethylenimine-coated Albumin Nanoparticles for BMP-2 Delivery. Biotechnol. Prog..

[22] R Development Core Team (2008). R: A Language and Environment for Statistical Computing.

[23] Merodio M., Arnedo A., Renedo M.J., Irache J.M. (2001). Ganciclovir-loaded albumin nanoparticles: Characterization and in vitro release properties. Eur. J. Pharm. Sci..

[24] Jahanban-Esfahlan A., Dastmalchi S., Davaran S. (2016). A simple improved desolvation method for the rapid preparation of albumin nanoparticles. Int. J. Biol. Macromol..

[25] Jiang J., Oberdörster G., Biswas P. (2009). Characterization of size, surface charge, and agglomeration state of nanoparticle dispersions for toxicological studies. J. Nanopart. Res..

[26] Borm P.J.A., Robbins D., Haubold S., Kuhlbusch T., Fissan H., Donaldson K., Schins R., Stone V., Kreyling W., Lademann J. (2006). The potential risks of nanomaterials: A review carried out for ECETOC. Part. Fibre Toxicol..

[27] Renwick L.C., Donaldson K., Clouter A. (2001). Impairment of alveolar macrophage phagocytosis by ultrafine particles. Toxicol. Appl. Pharmacol..

[28] Hoshino A., Fujioka K., Oku T., Suga M., Sasaki Y.F., Ohta T., Yasuhara M., Suzuki K., Yamamoto K. (2004). Physicochemical properties and cellular toxicity of nanocrystal quantum dots depend on their surface modification. Nano Lett..

[29] Qiu Y., Park K. (2012). Environment-sensitive hydrogels for drug delivery. Adv. Drug Deliv. Rev..

[30] Prajapati R., Gontsarik M., Yaghmur A., Salentinig S. (2019). pH-responsive nano-self-assemblies of the anticancer drug 2-Hydroxyoleic acid. Langmuir.

[31] Tannock I.F., Rotin D. (1989). Acid pH in tumors and its potential for therapeutic exploitation. Cancer Res..

[32] Catanzaro G., Curcio M., Cirillo G., Spizzirri U.G., Besharat Z.M., Abballe L., Vacca A., Iemma F., Picci N., Ferretti E. (2017). Albumin nanoparticles for glutathione-responsive release of cisplatin: New opportunities for medulloblastoma. Int. J. Pharm..

[33] Kennedy L., Sandhu J.K., Harper M.E., Cuperlovic-culf M. (2020). Role of glutathione in cancer: From mechanisms to therapies. Biomolecules.

[34] Estrela J.M., Ortega A., Obrador E. (2006). Glutathione in cancer biology and therapy. Crit. Rev. Clin. Lab. Sci..

[35] Ballatori N., Krance S.M., Notenboom S., Shi S., Tieu K., Hammond C.L. (2009). Glutathione dysregulation and the etiology and progression of human diseases. Biol. Chem..

[36] Curcio M., Blanco-Fernández B., Costoya A., Concheiro A., Puoci F., Alvarez-Lorenzo C. (2015). Glucose cryoprotectant affects glutathione-responsive antitumor drug release from polysaccharide nanoparticles. Eur. J. Pharm. Biopharm..

[37] KIPP J. (2004). The role of solid nanoparticle technology in the parenteral delivery of poorly water-soluble drugs. Int. J. Pharm..

[38] Oncul S., Ercan A. (2017). Discrimination of the effects of doxorubicin on two different breast cancer cell lines on account of multidrug resistance and apoptosis. Indian J. Pharm. Sci..

[39] Mo Y., Barnett M.E., Takemoto D., Davidson H., Kompella U.B. (2007). Human serum albumin nanoparticles for efficient delivery of Cu, Zn superoxide dismutase gene. Mol. Vis..

[40] Lv L., An X., Li H., Ma L. (2016). Effect of miR-155 knockdown on the reversal of doxorubicin resistance in human lung cancer A549/dox cells. Oncol. Lett..

[41] Wagh J., Patel K.J., Soni P., Desai K., Upadhyay P., Soni H.P. (2015). Transfecting pDNA to E. coli DH5α using bovine serum albumin nanoparticles as a delivery vehicle. J. Biol. Chem. Lumin..

[42] McGregor D., Bolt H., Cogliano V., Richter-Reichhelm H.B. (2006). Formaldehyde and glutaraldehyde and nasal cytotoxicity: Case study within the context of the 2006 IPCS human framework for the analysis of a cancer mode of action for humans. Crit. Rev. Toxicol..

[43] Albanese A., Tang P.S., Chan W.C.W. (2012). The effect of nanoparticle size, shape, and surface chemistry on biological systems. Annu. Rev. Biomed. Eng..

[44] Bar-On O., Shapira M., Hershko D.D. (2007). Differential effects of doxorubicin treatment on cell cycle arrest and Skp2 expression in breast cancer cells. Anticancer Drugs.

[45] Troester M.A., Hoadley K.A., Sørlie T., Herbert B.S., Børresen-Dale A.L., Lønning P.E., Shay J.W., Kaufmann W.K., Perou C.M. (2004). Cell-type-specific responses to chemotherapeutics in breast cancer. Cancer Res..

[46] Elzoghby A.O., Samy W.M., Elgindy N.A. (2012). Albumin-based nanoparticles as potential controlled release drug delivery systems. J. Control Release.

