# Supplementary material for: Albumin-Based Nanoparticles for the Delivery of Doxorubicin in Breast Cancer
Source: Cancers (Basel). 2021 Jun 16;13(12):3011. doi: 10.3390/cancers13123011 (PMC8235501; doi:10.3390/cancers13123011)
Supplement: Supplementary file 1 [file cancers-13-03011-s001.zip › cancers-1264826-supplementary.pdf]

cancers

Supplementary

Albumin-Based Nanoparticles for the Delivery of Doxorubicin in Breast Cancer

Rama Prajapati, Eduardo G. Garrido and Álvaro Somoza*

Instituto Madrileño de Estudios Avanzados en Nanociencia (IMDEA Nanociencia), Faraday 9, 28049 Madrid, Spain

* Correspondence: e-mail alvaro.somoza@imdea.org; Tel.: +34-912-998-856

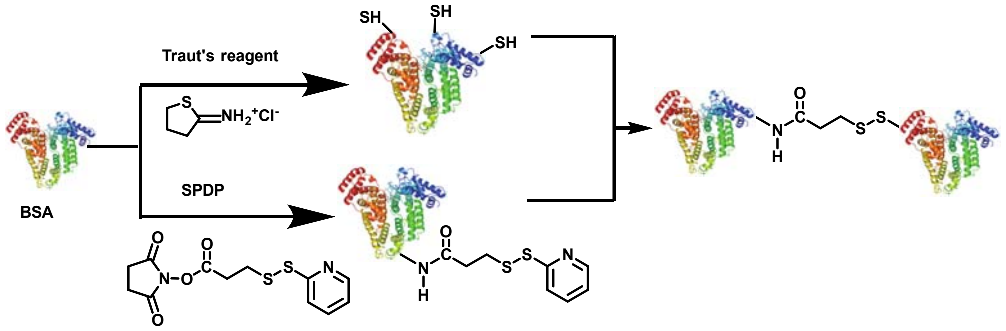

Figure S1. Surface modification of BSA with Traut's reagent and SPDP, followed by their conjugation through the formation of disulfide bonds to yield the corresponding BSA nanoparticles (ABN-SPDP). .

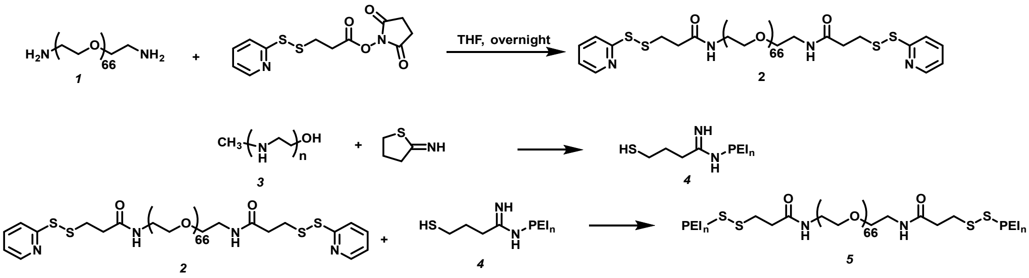

Figure S2. Schematic illustration of the preparation of PEI-PEG based polymer.

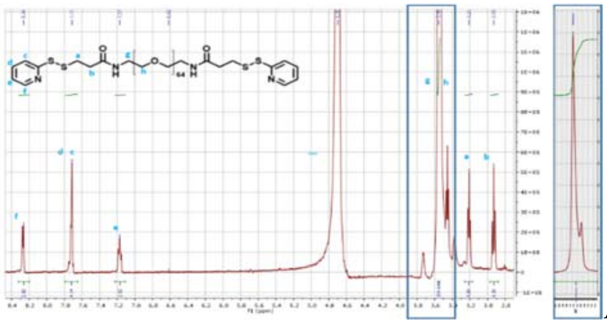

A

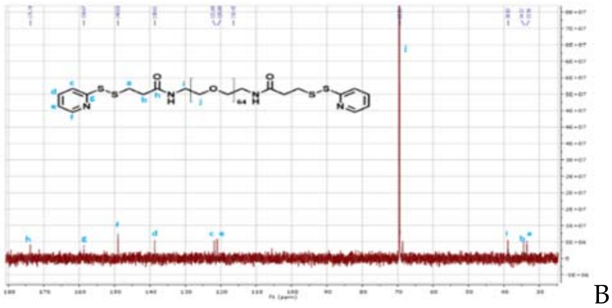

B

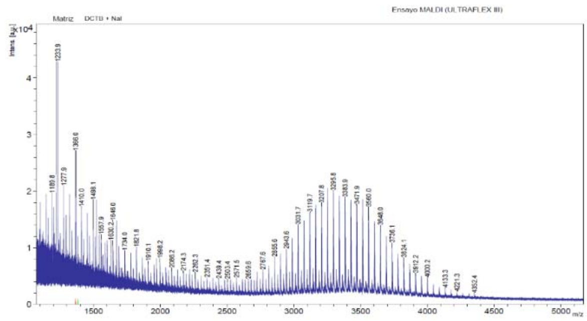

C

Figure S3. ^{1}H (A) ^{13}C NMR (B) and MS (C) of NH_2-PEG-NH_2 modified with SPDP.

A

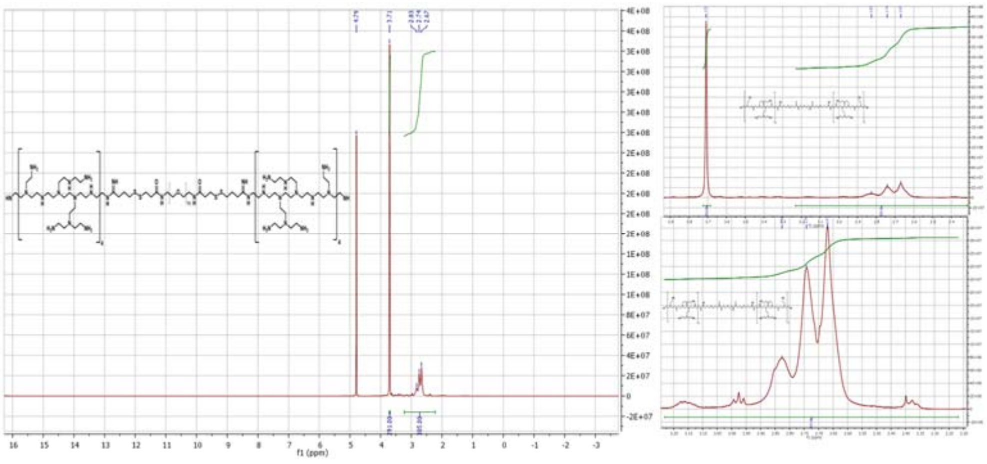

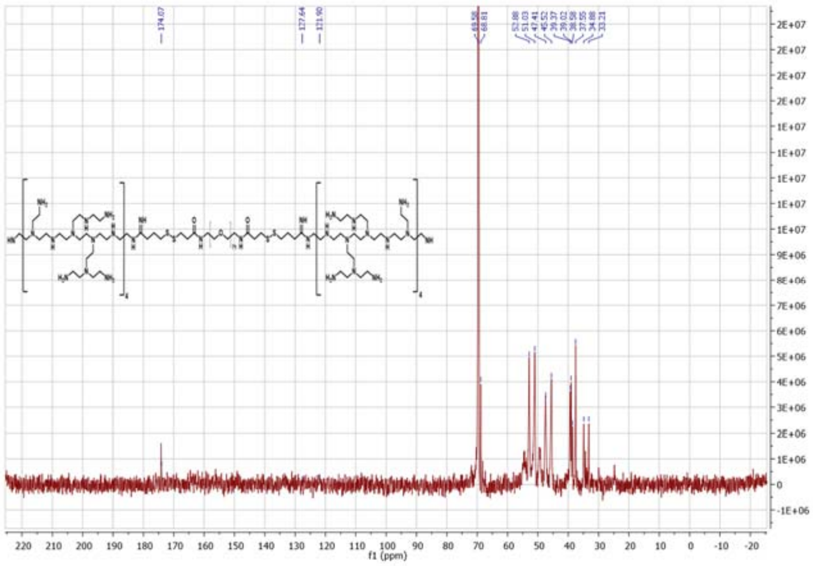

B

Figure S4. ^{1}H (A) and ^{13}C NMR (B) of PEI-PEG-PEI. In ^{1}H NMR, the PEG/PEI signal integrates in the ratio of 1:1.05, which corresponds approximately to the bi-functionalized PEG with PEI. In ^{13}C NMR, the characteristics peaks of the PEG at 70 ppm, and the peaks of PEI distributed between 50 and 35 ppm can be observed that correspond to the ethylene groups.

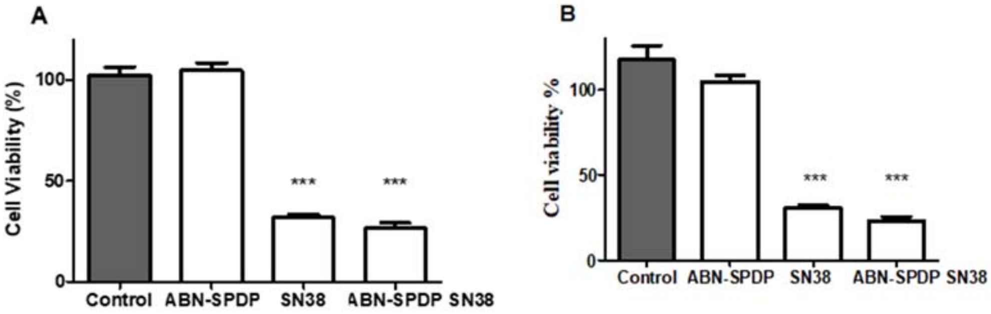

Figure S5. Cell viability assay with SN38 loaded ABN-SPDP in (A) MCF-7 and (B) MDA-MB-231 cells 48 hours post-treatment. Statistical analysis was performed using one-way ANOVA Tukey's test (*** *p*-value < 0.0001).

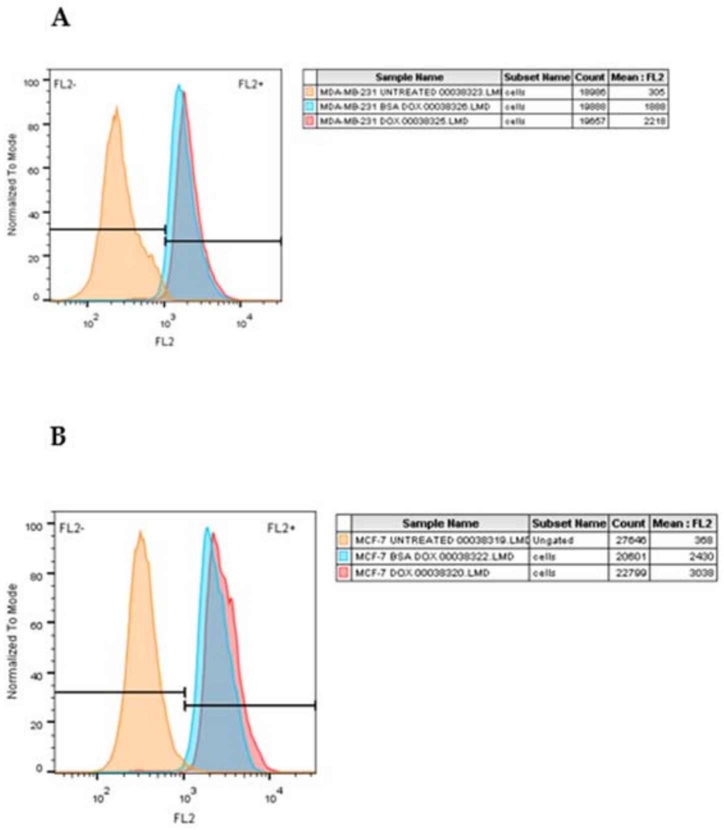

Figure S6. Quantification of fluorescence of Dox using flow cytometry in MDA-MB-231 (A) and MCF-7 (B) cells 24 hours post-treatment.

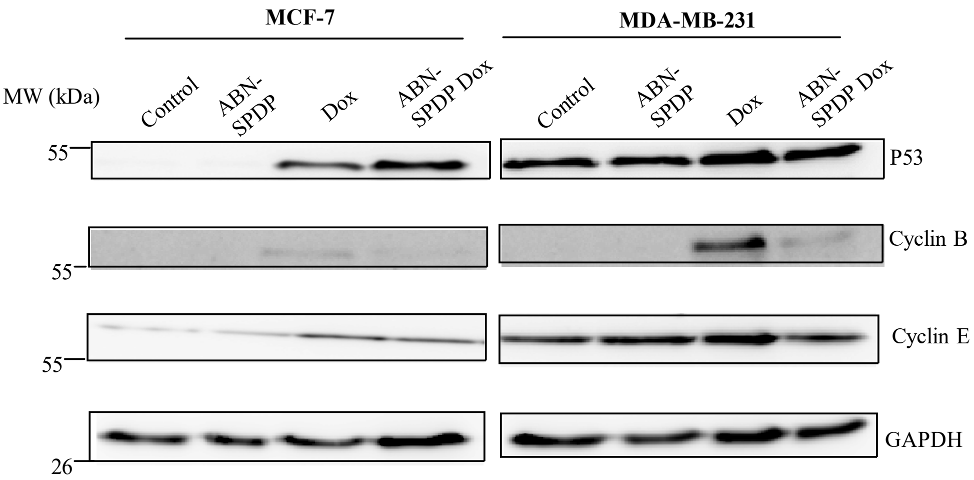

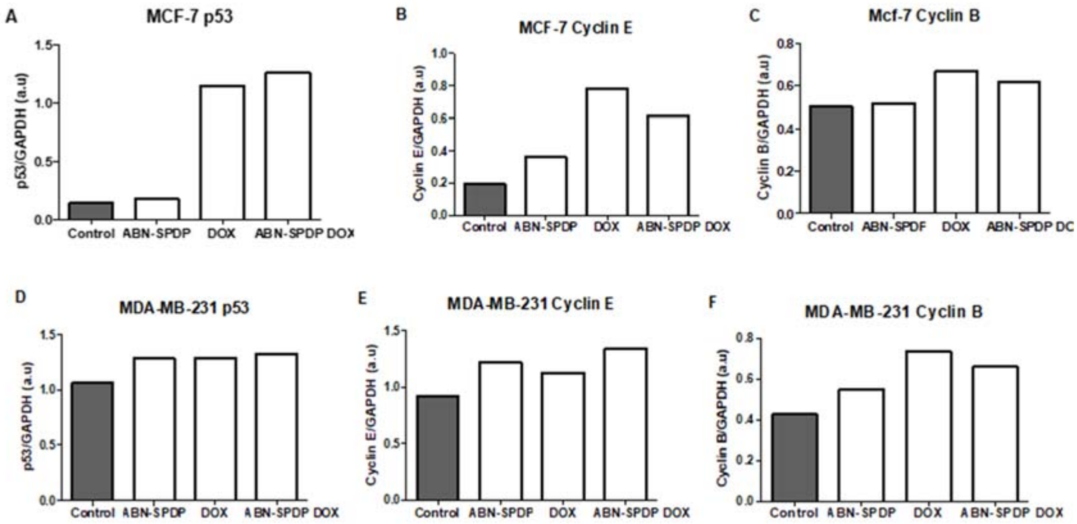

Figure S7. Western blot assay to assess the effect of Dox loaded ABN-SPDP on the expression of P53 and cyclins in MCF-7 and MDA-MB-231 cells.

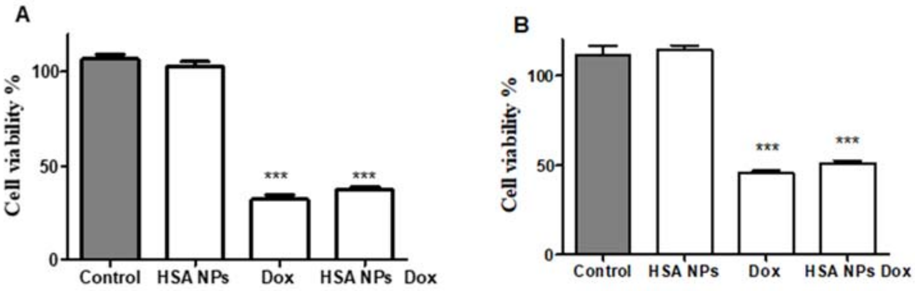

Figure S8. Cell viability assay with Dox loaded HSA NPs in (A) MCF-7 and (B) MDA-MB-231 cells 48 hours post-treatment. Statistical analysis was performed using one-way ANOVA Tukey's test (*** p-value < 0.0001).

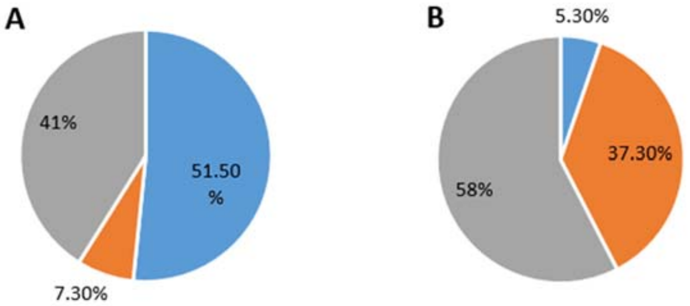

Figure S9. Percentage of cells in different phases of the cell cycle in (A) MCF-7 and (B) MDA-MB-231 cells treated with Dox-loaded HSA NPs. Blue: cells in G0/G1 phase; orange: cells in S phase; grey: cells in G2/M phase.
